# Behind the Curtain: A Pediatric Dermoid Tumor Masquerading as Craniopharyngioma

**DOI:** 10.7759/cureus.114113

**Published:** 2026-08-07

**Authors:** Annika Stückrath, Francisco Rivera, Pablo Negri

**Affiliations:** 1 Medicine, Universidad Maimónides, Buenos Aires, ARG; 2 Neurological Surgery, Barrow Neurological Institute, St. Joseph’s Hospital and Medical Center, Phoenix, USA; 3 Neurological Surgery, Hospital General de Agudos Dr. Juan A. Fernández, Buenos Aires, ARG

**Keywords:** craniopharyngioma, craniotomy, dermoid cyst, pediatric neurosurgery, suprasellar tumor

## Abstract

Dermoid cysts are rare, benign congenital tumors arising from ectodermal remnants that become trapped during neural tube closure. They frequently occur in midline structures such as the sellar region, where proximity to critical neurovascular structures creates diagnostic and surgical challenges. Craniopharyngiomas, in contrast, are benign epithelial tumors originating from Rathke's pouch remnants, and patients often present with visual deficits, panhypopituitarism, and hydrocephalus. We report the case of a 13-year-old female patient with progressive vision loss whose initial imaging suggested a craniopharyngioma but whose definitive pathology revealed a dermoid cyst. The patient presented with superior temporal quadrantanopia in the right eye and temporal hemianopia in the left eye. Magnetic resonance imaging (MRI) demonstrated a lesion in the sellar region extending into the suprasellar space. Surgical resection was performed through a right frontotemporal craniotomy, and complete resection was achieved. Although the clinical and radiological features suggested a craniopharyngioma, the lesion proved to be an atypical dermoid cyst. The patient remained neurologically stable postoperatively, with persistent visual impairment attributable to irreversible damage caused by tumor compression. Dermoid cysts in the sellar region may mimic craniopharyngiomas. Accurate diagnosis and careful surgical planning are essential given the proximity of these lesions to critical neurovascular structures. This case highlights the diagnostic overlap between dermoid cysts and adamantinomatous craniopharyngiomas in the pediatric population and the importance of establishing the correct diagnosis before surgery.

## Introduction

Craniopharyngiomas comprise 1.2%-4% of intracranial tumors in children and frequently arise in the sellar and suprasellar region [1]. These are rare, benign tumors derived from Rathke's pouch remnants. They are classified into two subtypes: adamantinomatous craniopharyngioma (ACP), the most common form, which affects children and adolescents and is characterized by cystic components and calcifications, often causing visual and endocrine dysfunction because of its proximity to the optic chiasm and pituitary gland, and whose cystic nature makes surgical resection challenging, and papillary craniopharyngioma (PCP), which primarily affects adults, is more solid with fewer cystic features and calcifications, presents more insidiously, and is more accessible for resection but prone to recurrence [2]. Intracranial dermoid cysts, on the other hand, are rare congenital tumors comprising less than 1% of all intracranial neoplasms [3]. Craniofacial dermoid cysts account for approximately 7% of all dermoids, with an incidence ranging between 0.03% and 0.14% [4]. They arise from ectodermal cell entrapment during neural tube closure and may remain asymptomatic. Clinical presentation varies according to cyst location; in an analysis of available case reports by El-Bahy et al., headache was the most common symptom (32.6%), followed by seizures (26.5%), cerebral ischemia with sensory and/or motor hemisyndrome (16.3%), and aseptic meningitis (8.2%) [5]. Rupture can result in hydrocephalus or chemical meningitis due to fat droplets in the subarachnoid space [6]. Intracranial dermoid cysts may mimic ACP in both clinical presentation and imaging, complicating the diagnosis [7]. Preoperative differentiation between these entities carries substantive operative and prognostic consequences: gross total resection is curative in dermoid cysts yet frequently unattainable in ACP; intraoperative cyst rupture with consequent chemical meningitis must be anticipated in the former but not the latter; and the two diagnoses entail materially different requirements for long-term endocrine and radiological surveillance. Reports describing this diagnostic overlap in the pediatric sellar region remain limited, and criteria for distinguishing these lesions preoperatively have yet to be clearly established. Therefore, we present the case of a 13-year-old girl with progressive visual loss and a suprasellar mass that was initially presumed to be an ACP but proved on histopathology to be a dermoid cyst, illustrating the diagnostic difficulty these lesions pose and the consequences of misclassification.

## Case presentation

**Figure 1:**
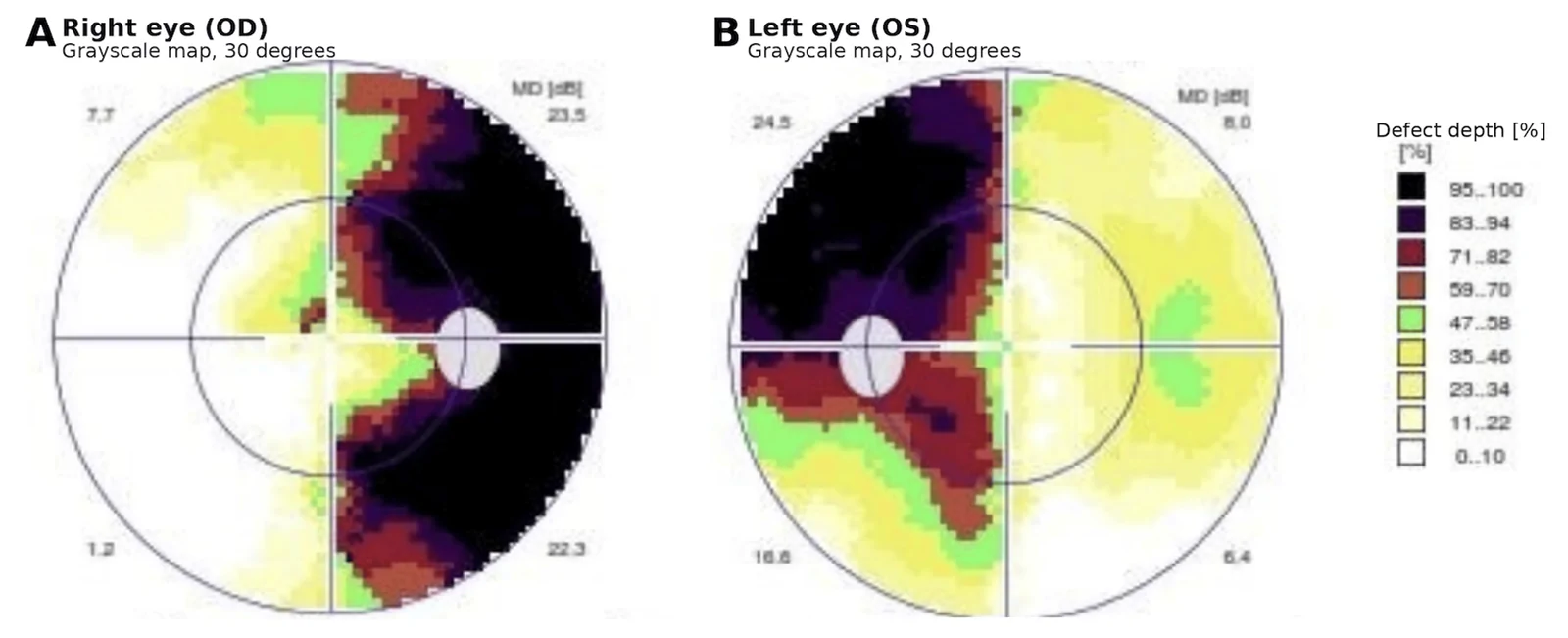
Octopus visual field testing (A) Right eye demonstrating superior temporal quadrantanopia. (B) Left eye demonstrating temporal hemianopia.

**Figure 2:**
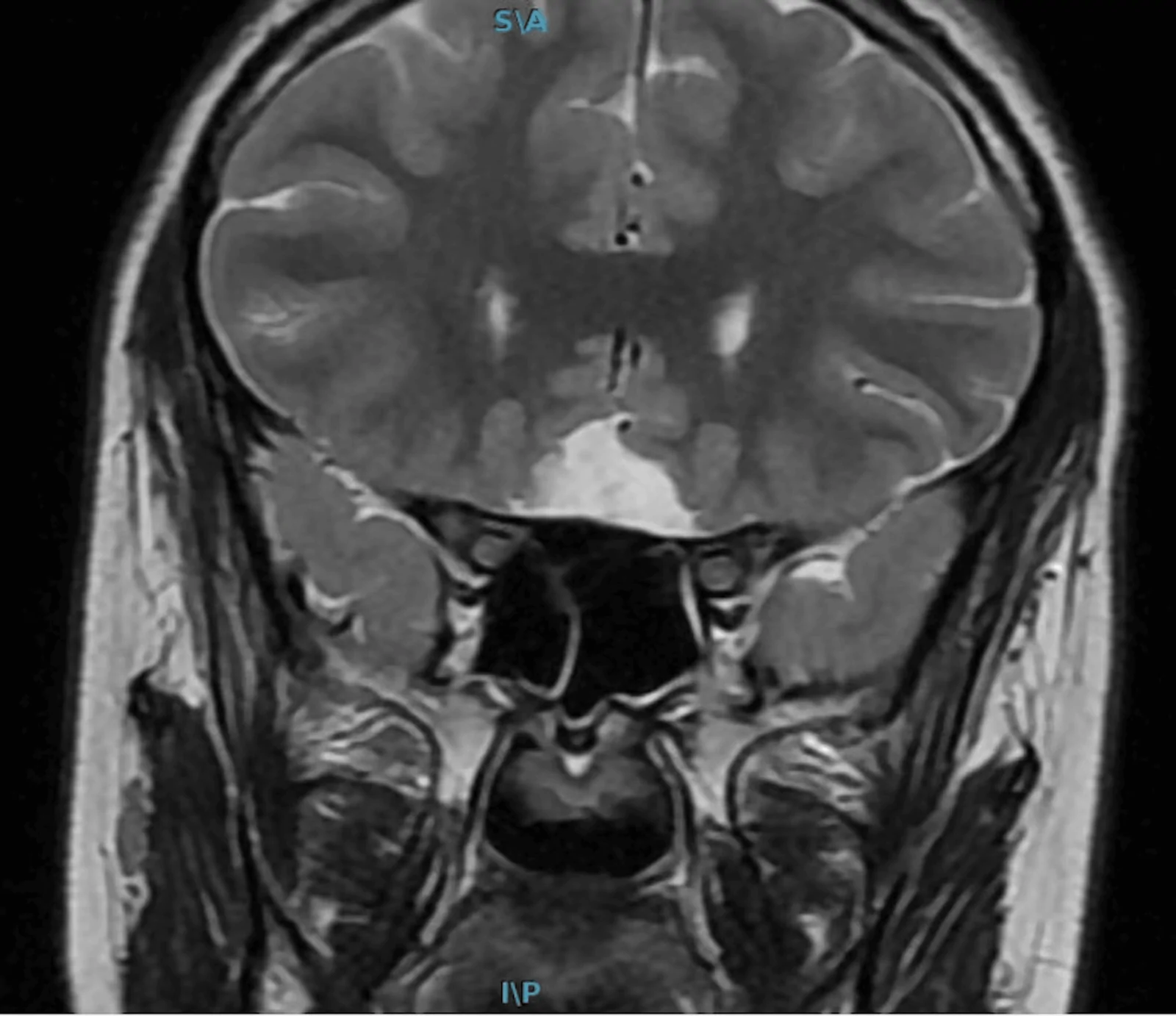
Coronal T2-weighted MRI of the sellar and suprasellar region A well-defined, hyperintense cystic lesion is seen in the sellar and suprasellar region, with mass effect on the optic chiasm. MRI: magnetic resonance imaging

**Figure 3:**
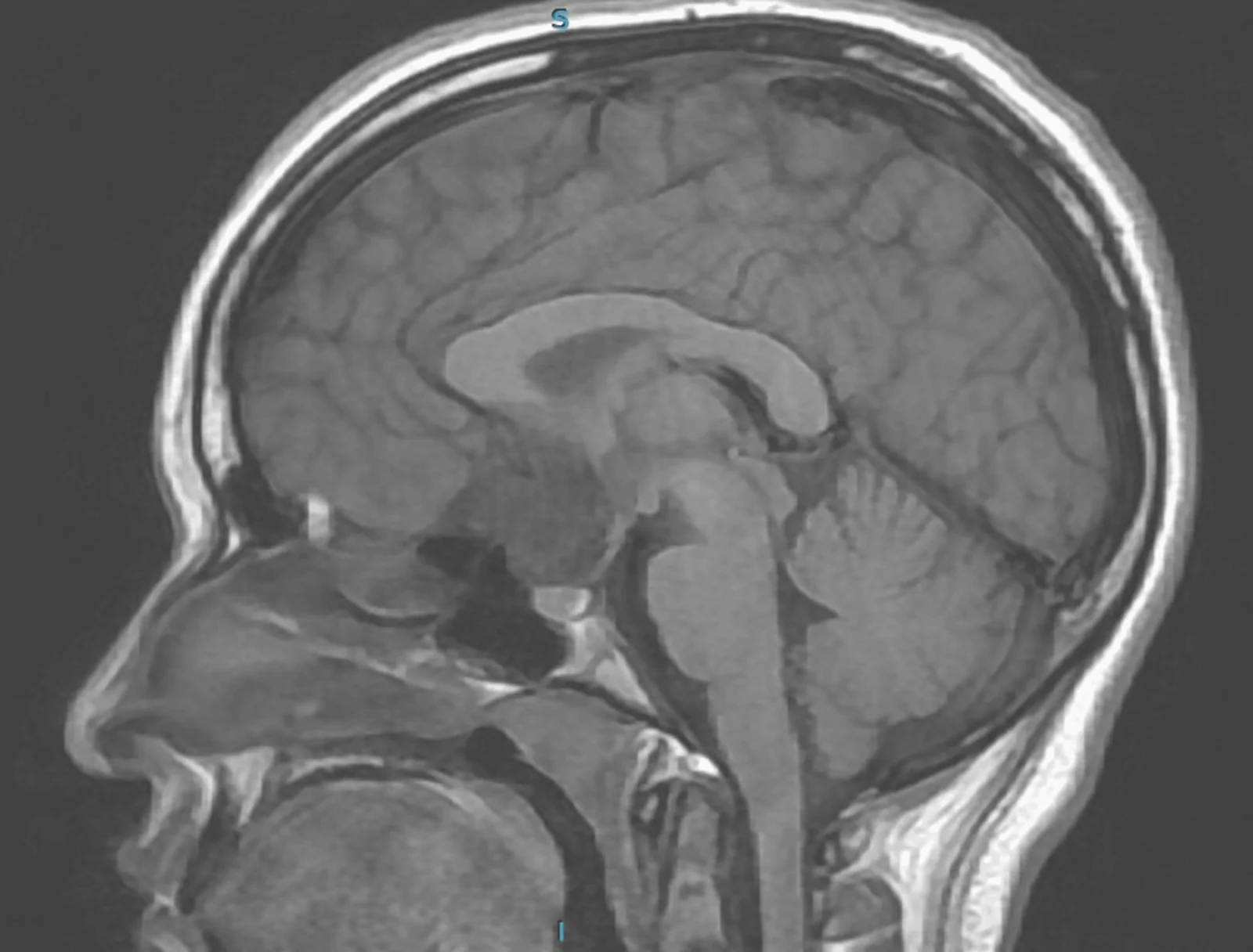
Sagittal T1-weighted MRI of the sellar and suprasellar region A well-defined, hypointense cystic lesion causes superior displacement of the optic chiasm and compression of the infundibulum. MRI: magnetic resonance imaging

A 13-year-old girl presented to the ophthalmology clinic for a routine check-up. On examination, she was found to have superior temporal quadrantanopia in the right eye (Figure 1A) and temporal hemianopia in the left eye (Figure 1B). Given the visual findings, we performed a T1-weighted, T2-weighted, and post-contrast T1-weighted brain magnetic resonance imaging (MRI) with axial, coronal, and sagittal planes. Unfortunately, diffusion-weighted imaging (DWI) and magnetic resonance (MR) spectroscopy were not available at our institution. The MRI demonstrated a suprasellar lesion measuring 28 x 30 x 28 mm, extending into the anterior cranial fossa as well as into both cavernous sinuses, encasing the carotid arteries. The lesion exhibited features classically associated with craniopharyngiomas: heterogeneous borders, hypointensity on T1-weighted images, and hyperintensity on T2-weighted images, with no fat-fluid level and no contrast enhancement (Figures 2, 3). The optic chiasm was displaced superiorly, and the infundibulum was compressed, without associated hydrocephalus.

Additional endocrinological studies were ordered to evaluate pituitary-hypothalamic function, including luteinizing hormone (LH), follicle-stimulating hormone (FSH), prolactin, calcium, phosphorus, magnesium, and tumor markers. All results were within normal limits except for plasma cortisol, which was low (4.01 µg/dL), suggesting a central adrenocorticotropic hormone (ACTH) deficiency consistent with secondary adrenal insufficiency, likely due to pituitary dysfunction from the mass effect of the lesion. Because of the low cortisol level and the risk of acute adrenal insufficiency in the perioperative period, intravenous hydrocortisone was administered (100 mg preoperatively, followed by 25 mg every six hours for 48 hours). The patient underwent surgery for visual field deficits and compression of the optic apparatus, and risk of neurological compromise.

**Figure 4:**
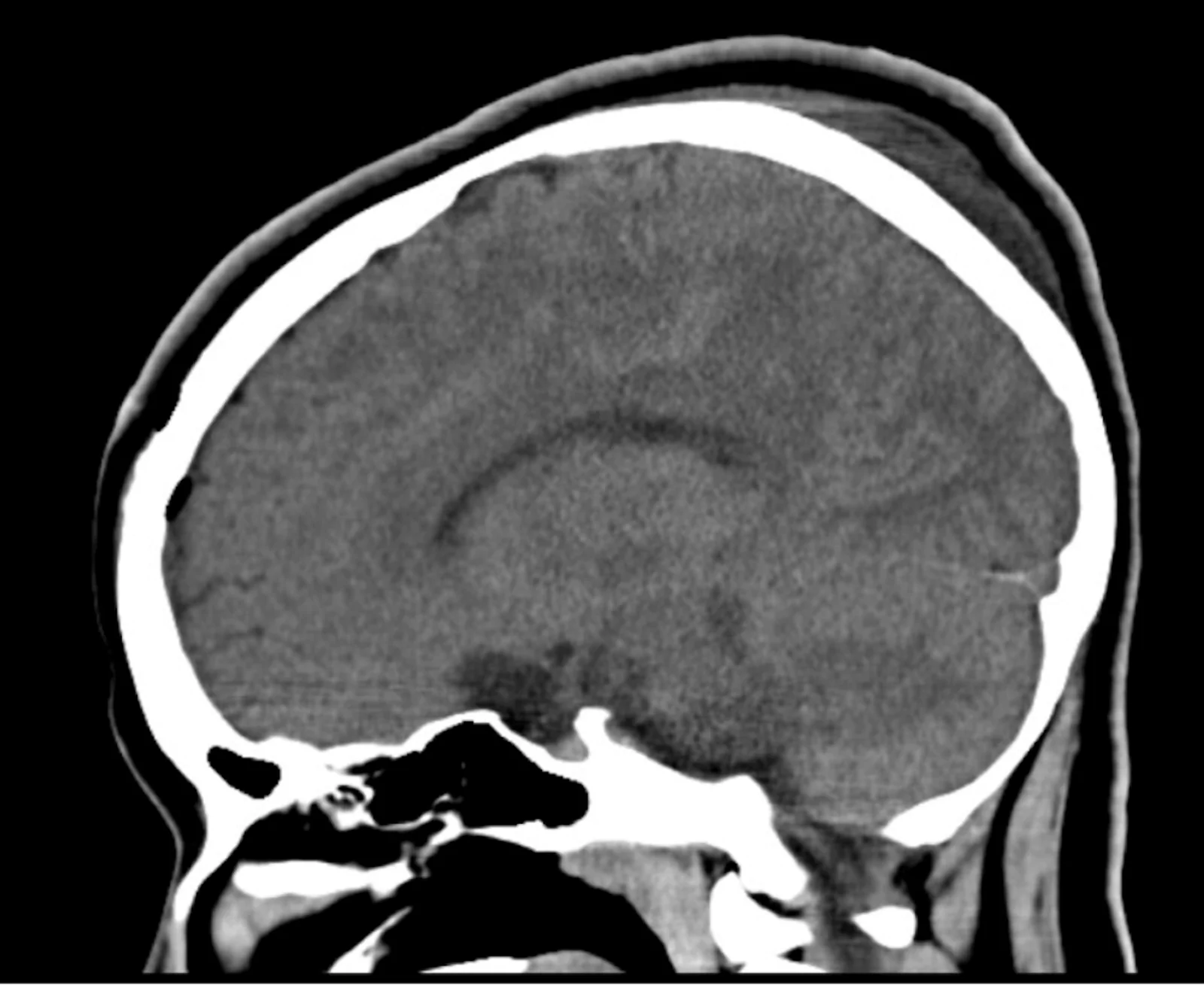
Postoperative sagittal CT of the head without contrast Imaging obtained after right frontotemporal craniotomy and resection of the lesion. CT: computed tomography

With the patient in the supine position and under general anesthesia, a right temporofrontal incision was made, followed by the creation of a myocutaneous flap. A right frontotemporal craniotomy was performed to access the sellar region through the floor of the anterior cranial fossa. The cyst appeared as a soft, whitish mass with a heterogeneous consistency, poorly defined margins, and an irregular interface with the surrounding brain parenchyma. Upon excision, it yielded a viscous, yellowish sebaceous material characteristic of dermoid cysts, composed of desquamated epithelial cells, keratin, and lipid-rich debris. Decompression of the optic nerves and optic chiasm was successfully achieved. Given the intraoperative appearance of the lesion, a dermoid cyst was suspected. Histopathologic examination revealed a cyst lined by stratified squamous epithelium containing mature skin appendages and abundant lamellated keratin, with positive immunohistochemical staining for CK1, CK10, and CK6. These findings were consistent with a dermoid cyst. The patient tolerated the procedure well and was monitored postoperatively in the intensive care unit. No postoperative complications occurred. At discharge, endocrine function was stable, with no signs of hypopituitarism or diabetes insipidus (Figure 4). Written informed consent for publication was obtained from the patient's parents.

## Discussion

This case illustrates the clinical mimicry between craniopharyngiomas and dermoid cysts, the disabling impact of suprasellar tumors in children, and the need for diagnostic precision before surgery. It underscores the challenges of differentiating these lesions preoperatively and highlights the implications of diagnostic uncertainty for surgical decision-making and long-term patient outcomes.

The paradigm of benignity in suprasellar dermoid cysts

Dermoid cysts are traditionally regarded as benign, slow-growing congenital lesions with well-defined borders and minimal invasiveness. In our case, however, the critical location of the lesion and its mass effect on adjacent neurovascular structures led to severe and potentially irreversible symptoms, despite the absence of true invasion of the cavernous sinuses. This degree of local extension challenges the conventional understanding of dermoid cysts as non-aggressive entities and raises the possibility that a subset may exhibit a more infiltrative pattern, similar to craniopharyngiomas or even low-grade neoplasms [7,8]. Our case demonstrated extensive local extension behavior for a dermoid cyst located in the suprasellar region. Because the lesion presented in an atypical form, craniopharyngioma was suspected first, given its frequency in the pediatric population. Aggressive dermoid cysts pose an additional surgical challenge: intraoperative rupture. Rupture can cause chemical meningitis, hydrocephalus, and a significant inflammatory response through the release of sebaceous, lipid-rich contents into the subarachnoid space [9]. Preoperative recognition of an atypical dermoid cyst is therefore crucial for surgical planning, choice of approach, and perioperative care. While the literature on aggressive dermoid cysts remains scarce, previous reports have described cases in which rupture led to chemical meningitis, hydrocephalus, or an inflammatory response mimicking neoplastic infiltration [4]. Liu et al. have shown that long-term monitoring with serial MRI and clinical examination in patients with extensive disseminated fat particles has not demonstrated progression or migration of the fat, nor new neurological deterioration [6]. In those cases, medical management is indicated for symptom control. This case raises the question of whether dermoid cysts should be stratified according to their radiological and intraoperative behavior, which could influence surgical planning and postoperative surveillance strategies.

The illusion of diagnosis: pattern recognition bias in neurosurgery

The radiological similarity between craniopharyngiomas and dermoid cysts led to an initial misdiagnosis in this case, reflecting a cognitive bias in which well-established imaging patterns drive diagnostic certainty. Craniopharyngiomas, particularly the adamantinomatous subtype, often present with cystic components, calcifications, and heterogeneous enhancement, features that are also observed in suprasellar dermoid cysts [10]. Given that 58.3% of atypical epidermoid cysts are misdiagnosed preoperatively, as reported by Ren et al., our findings reinforce the need for a systematic, multimodal approach to the differentiation of sellar region tumors [11]. Advanced imaging techniques such as DWI and MR spectroscopy may help distinguish these lesions, as dermoid cysts typically exhibit restricted diffusion due to keratin and lipid content, whereas ACPs show variable diffusion restriction depending on their cystic and solid components [10]. However, reliance on imaging alone remains insufficient. Intraoperative frozen section analysis prior to definitive resection may serve as a critical adjunct to prevent unnecessary aggressive surgery for benign lesions. Pediatric dermoid cysts and ACPs present a significant diagnostic and surgical challenge given their midline suprasellar localization, overlapping clinical syndromes, and radiological mimicry. While dermoid cysts are benign ectodermal inclusions with well-defined borders and minimal mass effect, ACPs may exhibit local infiltration, frequently encasing the optic chiasm, hypothalamus, and pituitary stalk, which complicates safe resection. Surgically, gross total resection is curative for dermoid cysts, but intraoperative rupture carries a risk of chemical meningitis, requiring prompt cerebrospinal fluid diversion and anti-inflammatory management. In contrast, ACPs carry a high recurrence rate of approximately 30%-50%, making subtotal resection with adjuvant radiotherapy the preferred strategy when hypothalamic invasion precludes total excision [12]. Neuroimaging plays a crucial role in differentiation: dermoid cysts exhibit T1 hyperintensity, lack enhancement, and may contain fat-fluid levels on fluid-attenuated inversion recovery sequences, whereas ACPs demonstrate solid-cystic heterogeneity, coarse calcifications, and contrast-enhancing solid components. However, the current literature lacks definitive imaging markers to reliably distinguish these entities, leaving the neurosurgeon reliant on intraoperative findings and histopathological confirmation. Emerging modalities, including MR spectroscopy, intraoperative MRI, and advanced endoscopic techniques, may refine preoperative diagnosis and improve the extent of resection in these complex cases [13].

Surgical indications and rationale

Surgical intervention is indicated in patients who, at diagnosis or during follow-up, present with visual field deficits, ophthalmoplegia, or other neurological impairment secondary to lesion compression; frank contact of the tumor with the optic nerves or optic chiasm; pituitary apoplexy with visual compromise; or hypersecretory pituitary incidentalomas other than prolactinomas [14]. Kucera et al. recommend surgical management only in the presence of mass effect associated with severe neurological deficits, emphasizing complete resection of both the cyst capsule and its contents to prevent recurrence in intact lesions, with meticulous dissection from adjacent neurovascular structures as the primary surgical objective [15]. Similarly, Lynch et al. advocate total tumor resection as the optimal surgical strategy, particularly for lesions compressing the brainstem or optic chiasm. Using careful microsurgical technique and progressive debulking to preserve arachnoid planes, they reported high cure rates with low morbidity in a retrospective review of 33 consecutive patients (14 men and 19 women; mean age at surgery 37.9 years) with pathologically confirmed intracranial dermoid and epidermoid tumors, with a mean follow-up of 7.2 years [16]. In contrast, Singla and Kapoor questioned whether surgical intervention is mandatory in all cases of rupture, suggesting careful patient selection in asymptomatic or low-risk cases. They acknowledge, however, that total resection reduces the risk of intraventricular dissemination, although the procedure remains technically challenging because of the widespread dispersion of cyst contents within the subarachnoid spaces [17].

The underestimated impact of visual loss in pediatric sellar tumors

In pediatric patients, visual deficits secondary to suprasellar tumors extend beyond neurological morbidity and significantly affect long-term quality of life [18]. Our patient exhibited persistent visual impairment despite successful tumor resection, highlighting the potentially irreversible consequences of prolonged optic chiasm compression. The likelihood of visual recovery is closely related to the duration and severity of preoperative compression. In a prospective observational study, Yoneoka et al. demonstrated that early morphological recovery of the optic chiasm after decompression is associated with favorable visual outcomes, whereas delayed intervention often results in limited improvement, underscoring the need for timely surgical management to prevent permanent deficits [19]. In a retrospective case series, Alswaina et al. reported that nearly half of pediatric brain tumor patients present with vision loss and optic atrophy at diagnosis, reinforcing the need for earlier detection and intervention [20]. While the primary goal of neurosurgical intervention in sellar region tumors has traditionally been gross total resection, this case emphasizes the need to re-evaluate surgical priorities. Given that a substantial proportion of children with craniopharyngiomas experience persistent visual deficits postoperatively [2], alternative strategies focusing on optic pathway decompression rather than maximal resection may warrant further exploration. Future research should investigate whether early intervention, adjunct therapies, or neuroprotective strategies can mitigate long-term visual disability in pediatric patients with suprasellar masses.

## Conclusions

This case reinforces the need for heightened vigilance and timely intervention in pediatric sellar lesions to prevent irreversible morbidity. The recognition of dermoid cysts with atypical local extension, the cognitive biases that influence preoperative diagnosis, and the enduring impact of visual loss warrant careful consideration in the management of these patients, in whom histopathological examination remains indispensable to the definitive diagnosis and the operative strategy is best individualized to the intraoperative findings. Future studies in larger series should explore improved diagnostic algorithms incorporating advanced imaging, intraoperative pathology, and functional outcome-based surgical strategies to optimize patient care.
